# Clinical Presentation and Percutaneous Treatment of Hemolytic Anemia Associated With Paravalvular Leak After TAVR

**DOI:** 10.1016/j.jaccas.2024.102748

**Published:** 2024-12-04

**Authors:** Matteo Sturla, Andrea Scotti, Antonella Millin, Julio Echarte-Morales, Guillaume Bonnet, Andrea Mignatti, Manaf Assafin, Leandro Slipczuk, Edwin Ho, Azeem Latib

**Affiliations:** a Montefiore-Einstein Center for Heart and Vascular Care, Montefiore Medical Center, Albert Einstein College of Medicine, Bronx, New York, USA; b Cardiovascular Research Foundation, New York, New York, USA; c Medico-Surgical Department, Haut-Lévêque Cardiological Hospital, Bordeaux University Hospital, Pessac, France

**Keywords:** hemolytic anemia, paravalvular leak, TAVR

## Abstract

Paravalvular leak (PVL) following transcatheter aortic valve replacement (TAVR) is an established complication, albeit rarely associated with hemolytic anemia. This report details 3 cases of significant hemolytic anemia attributed to TAVR-induced PVL, each with distinct clinical presentations and manifestations. These cases underscore the diverse and occasionally subtle clinical presentation of aortic PVL-associated hemolytic anemia. We explore the technical nuances and challenges of percutaneous PVL closure and emphasize the successful resolution of hemolytic anemia by using percutaneous PVL plugging with occluder devices. Through this case series, we encourage the awareness of PVL-associated hemolytic anemia in the differential diagnosis of patients presenting with new onset anemia after TAVR and demonstrate the efficacy of percutaneous closure techniques in resolving this serious condition.

## Visual Summary

**Figure:**
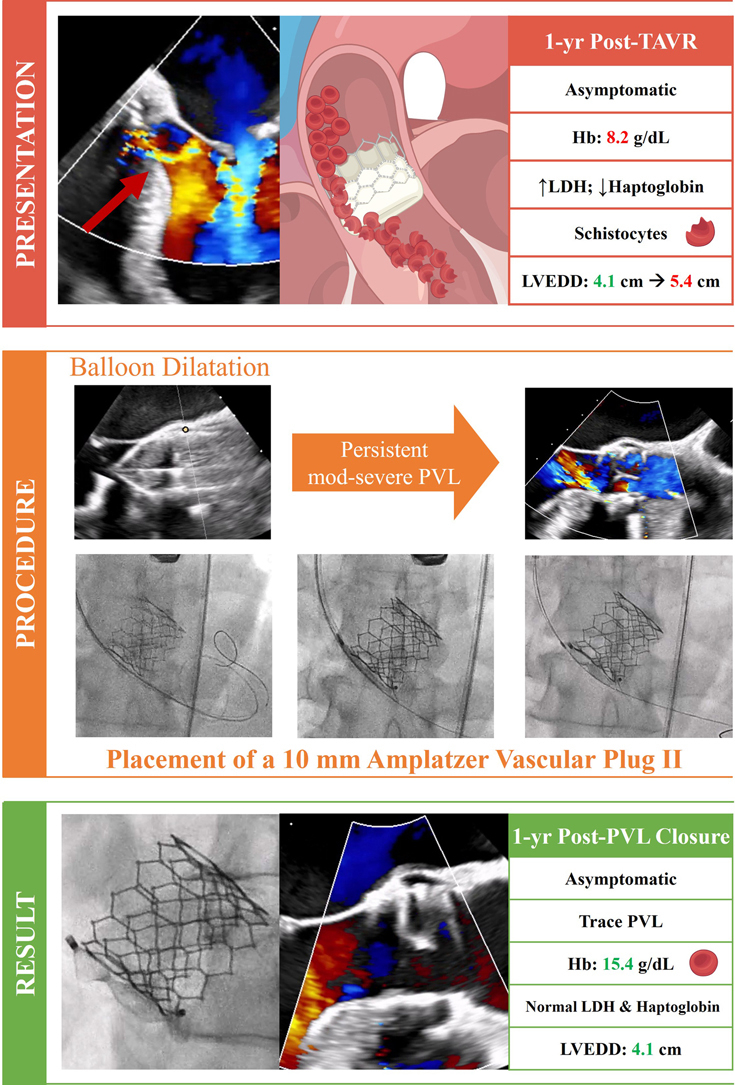

The advancement of transcatheter aortic valve replacement (TAVR) as a transformative approach for aortic stenosis (AS) is accompanied by the recognized complication of paravalvular leak (PVL). Moderate or greater PVL has been reported in approximately 12% of patients with first-generation valves, although this complication rate decreases to <5% with the latest iterations.^1^, ^2^, ^3^ Nevertheless, significant PVL is associated with increased mortality.^1^, ^2^, ^3^ Hemolytic anemia, resulting from mechanical shear stress on red blood cells as they pass through a PVL, is seldom reported post-TAVR. Rather, this phenomenon is more frequently associated with PVLs after mitral valve replacement. Through this series, we aimed to explore the clinical complexities, procedural characteristics, and positive impact of PVL closure in resolving hemolytic anemia in the context of TAVR procedures.

**Take-Home Messages**

• Recognizing cardiac prosthesis-related hemolytic anemia is crucial in patients who have undergone TAVR.
• Percutaneous closure of PVLs in TAVR devices is feasible but presents significant technical challenges.
• Successful aortic PVL closure can result in the resolution of hemolytic anemia, demonstrating significant clinical benefits.

## Case 1

A 77-year-old man presented to the emergency department from an ambulatory clinic after concerning laboratory findings despite his being functionally asymptomatic. Laboratory findings on admission revealed progressive anemia, with hemoglobin (Hb) decreasing to 8.2 g/dL from 15.5 g/dL 14 months earlier. Hematologic work-up demonstrated a strong hemolytic pattern, including elevated reticulocytes (4.2%), increased lactate dehydrogenase (LDH) levels (1,395 U/L), and depleted haptoglobin (<10 mg/dL). A peripheral blood smear revealed 2+ schistocytes.

The patient had a significant past medical history of hypertension, diabetes, chronic obstructive pulmonary disease, well-controlled human immunodeficiency virus infection, hepatitis B virus infection, and tuberculosis (The Society of Thoracic Surgeons [STS] score, 6.31%). One year previously, he underwent TAVR with a 26-mm Sapien 3 Ultra valve (Edwards Lifesciences) for severe symptomatic AS. Preprocedural computed tomography showed commissural calcifications of the right and noncoronary cusp commissures. Post-dilation reduced the PVL from severe to mild after valve implantation. At the time of discharge for the index TAVR, the patient’s Hb level was stable (15.3 g/dL). Transthoracic echocardiography (TTE) at 1-month post-TAVR showed that the PVL was stable, with mild to moderate severity.

Whereas TTE imaging showed left ventricular (LV) dilation with preserved function, transesophageal echocardiography (TEE) confirmed preserved bioprosthesis function with a moderate to severe PVL (jet vena contracta, 5.7 mm; and effective regurgitant orifice area, 0.37 cm^2^) (Video 1).

After a heart team discussion, percutaneous closure of the PVL was performed (Video 1). Initial balloon dilation with a 25-mm True Balloon (BD Life Sciences) showed persistent moderate to severe PVL, thus leading to escalation of percutaneous closure with a 10-mm Amplatzer Vascular Plug II (Abbott Vascular). By using a 6-F Destination sheath (Terumo), the distal disk of the plug was positioned on the ventricular side of the PVL. Then the sheath was slowly pulled back to deploy the rest of the device in the PVL orifice. TEE confirmed an excellent result, with only a trace residual PVL. The patient had 1-month, 6-month, and 1-year follow-ups post-PVL closure, with Hb increasing to 15.4 g/dL at 1 year and TTE showing a trace PVL, persistently stable.

## Case 2

A 69-year-old man, with a past medical history of hypertension, diabetes, end-stage renal disease treated with dialysis, pulmonary hypertension, and hypercholesterolemia (STS score, 10.3%), underwent TAVR using a 26-mm Sapien 3 Ultra valve for symptomatic AS in a bicuspid type 0 aortic valve. The patient underwent a successful procedure, with mild PVL evident on TTE, and was discharged without complications. However, 1 month later, he was readmitted with diffuse pruritus and reported pale stools and dark urine. On examination, jaundice and scleral icterus were evident. The patient was clinically euvolemic, adequately perfused, and in NYHA functional class I.

Laboratory findings revealed a significant drop in Hb since discharge, going from 10.2 to 7.9 g/dL, increased reticulocytes and LDH, depleted haptoglobin, and the presence of schistocytes on the peripheral blood smear. Laboratory findings revealed an unusual elevation in transaminases and predominantly direct hyperbilirubinemia (total bilirubin, 8.9 mg/dL; direct bilirubin, 6.3 mg/dL), atypical in intravascular hemolytic anemia cases where indirect hyperbilirubinemia typically prevails. This clinical and laboratory profile raised suspicions of concomitant cholelithiasis as a potential consequence of persistent hemolytic anemia. An abdominal ultrasound scan indicated mild common bile duct (CBD) dilatation without clear choledocholithiasis. Subsequent endoscopic retrograde cholangiopancreatography revealed a CBD stricture, and stent placement led to a decrease in bilirubin levels to 2.7 mg/dL. Anemia persisted, however, reaching even lower values of 7.2 g/dL.

**Figure 1:**
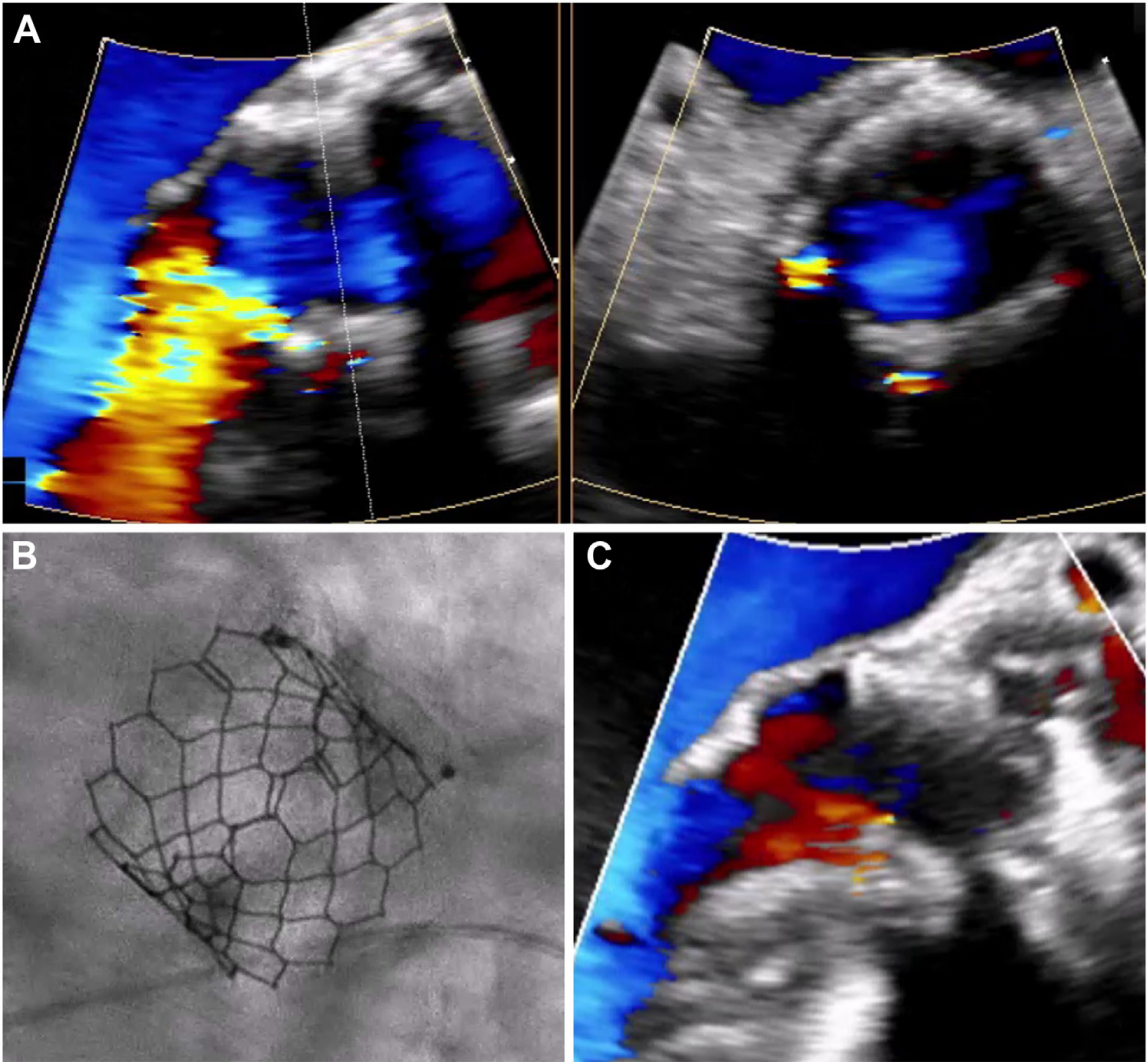
Case 2 Paravalvular Leak Evaluation and Final Result (A) Transesophageal evaluation of paravalvular leak evidencing a mild paravalvular leak. (B and C) Final angiographic and echocardiographic result after percutaneous plugging.

After ruling out alternative causes of anemia, TEE was performed to assess the PVL, the main suspected cause of the patient’s hemolytic anemia. It revealed a normal LV size with a normally functioning bioprosthesis and highlighted an eccentric, posteriorly directed, mild PVL (Figures 1A to 1C, Video 2).

Following heart team deliberation, the patient underwent PVL closure. The PVL was traversed with a straight-tip glide wire, which was exchanged for a Safari small. Post-dilatation with a 25-mm, then 26-mm True Balloon resulted in an unsatisfactory reduction of the PVL grade. Subsequently, a 7-mm Amplatzer Muscular VSD Occluder (Abbott Vascular) was deployed with the disks within the PVL tract, thereby reducing interaction with adjacent cardiac anatomy, mitigating the potential for hemolysis secondary to flow along the device, and resulting in a significant reduction in retrograde flow, as confirmed by intraprocedural TEE (Figures 1A to 1C, Video 2).

At 1-month follow-up, Hb levels increased to 11.6 g/dL, and hemolysis laboratory values normalized, aligning with the patient’s initial values and comorbidities. The echocardiographic follow-up confirmed the persistence of a good result with a trace PVL (Figures 1A to 1C, Video 2). At 3-year follow-up, laboratory values remained stable, with no evidence of hemolysis.

## Case 3

The case involved a 74-year-old patient with a past medical history of hypertension, diabetes, obesity, remote pulmonary embolism treated with warfarin, and a known thoracic aortic aneurysm. The patient had a decade-long history of smoldering immunoglobulin G lambda multiple myeloma, leading to stable and mild chronic anemia (Hb range, 11-12 g/dL), mild leukopenia, and chronic kidney disease (STS score, 11.0%).

Given his severe AS with a calcified type 1 bicuspid aortic valve, the patient underwent TAVR with a 23-mm Sapien 3 Ultra valve. Mild to moderate PVL was noticed after valve implantation, thus prompting post-dilation. First, the valve delivery system was used with an additional 2 mL of volume added to this delivery balloon; then, a 24-mm True Balloon was used, yielding a final postprocedural result of mild PVL. However, during the hospital stay, the patient developed hematuria and a significant drop in Hb from a preprocedural level of 12.8 g/dL down to 6.9 g/dL within 10 days, thereby necessitating blood transfusion. Concurrently, acute-on-chronic renal failure occurred, with his creatinine level rising from 1.5 to 2.8 mg/dL. Laboratory analysis suggested acute hemolytic anemia complicated by acute renal failure.

**Figure 2:**
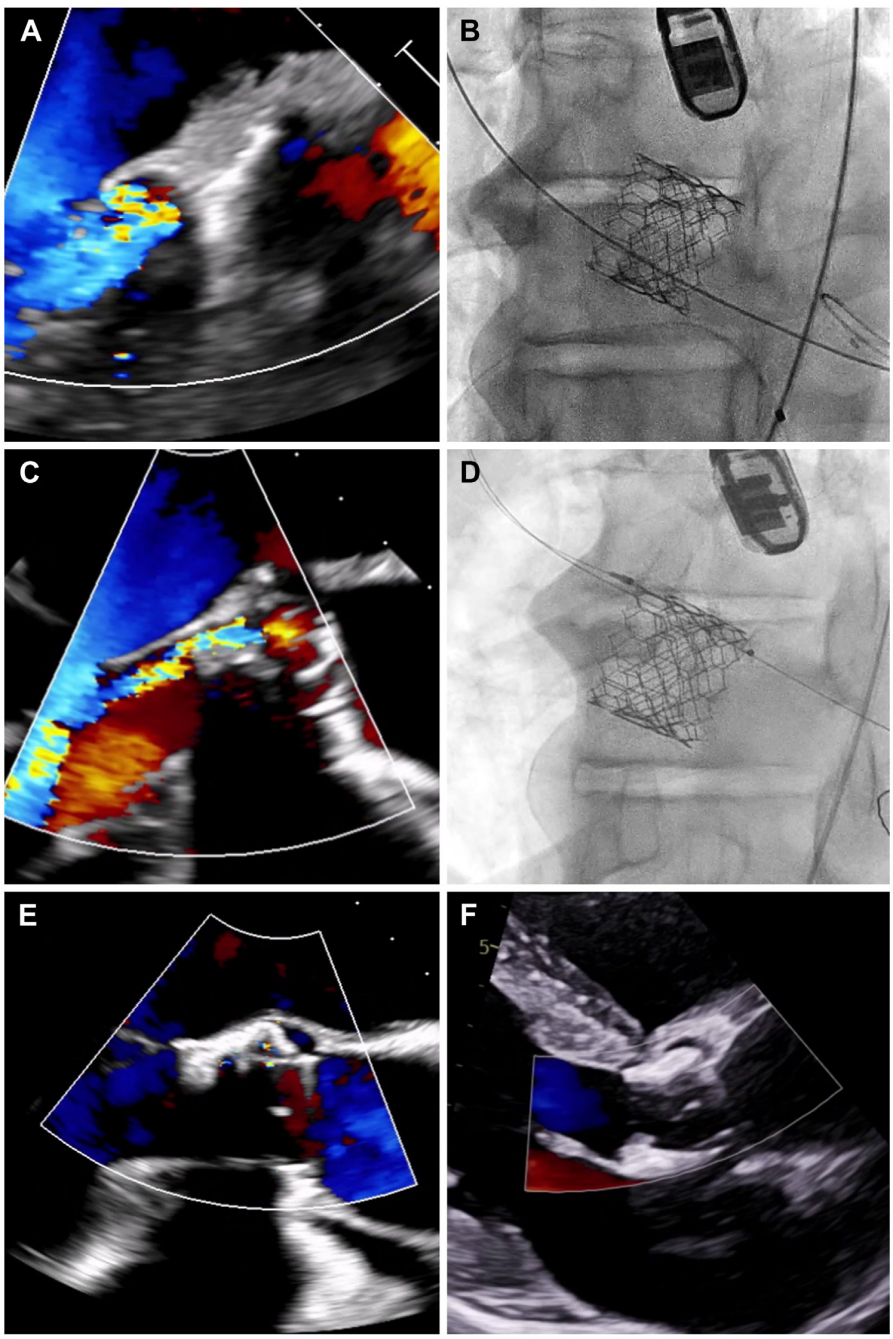
Case 3 Paravalvular Leak Evaluation and Final Result (A) Transesophageal echocardiographic evaluation of paravalvular leak after index transcatheter aortic valve replacement evidencing a mild paravalvular leak. (B) Final angiographic view after redo transcatheter aortic valve replacement in an attempt to resolve the paravalvular leak. (C) Transesophageal echocardiographic evaluation of a paravalvular leak after redo transcatheter aortic valve replacement evidencing a persistent significant paravalvular leak. (D) Angiographic result after percutaneous plugging. (E) Postprocedural transesophageal echocardiographic evaluation with only a trace paravalvular leak. (F) Follow-up transthoracic echocardiogram with no evidence of paravalvular leak.

Post-intervention day 1 TTE revealed a mean gradient of 19 mm Hg and moderate PVL characterized by 3 jets, with a total circumferential extent of >10% (Figures 2A to 2F, Video 3). To address these complications, the patient underwent a transcatheter aortic valve (TAV)-in-TAV procedure using a 23-mm Sapien 3 Ultra and post-dilated with a 23-mm True Balloon. Given the complexity of the PVL with multiple jets, it was determined that a second valve implantation would offer a more comprehensive solution by addressing the entire PVL pattern, rather than attempting individual closure of each jet with percutaneous devices, which may have been less effective and more technically challenging. More aggressive post-dilation was not possible because of severe aortic calcification. TEE confirmed a successful outcome, with minimal PVL. The patient experienced resolution of hematuria, and on discharge from the hospital, he had an Hb level of 8.3 g/dL.

Seven months later, however, he presented again with a report of fatigue and dyspnea on exertion. He had undergone regular cardiologic follow-up, and the echocardiographic findings remained unchanged, indicating normal prosthesis function and minimal PVL. Persistent hemolytic anemia was noted during routine laboratory examinations, with Hb values fluctuating between 7 and 8.5 g/dL. This necessitated multiple blood transfusions in addition to regular supplementation with iron, vitamin B_12_, and erythropoietin. However, given the patient’s active multiple myeloma, it was unclear which process contributed to his symptoms and persistent anemia. After multidisciplinary consultation with the hematology/oncology team, a decision was made to address the issue by closing the PVL (Figures 2A to 2F, Video 3). The patient underwent a successful closure procedure using a 7-mm Amplatzer Vascular Plug II. TEE confirmed the correct positioning of the device, with no impact on the anterior mitral valve leaflet and only trace paravalvular aortic regurgitation. The patient was discharged after 1 day.

Subsequent follow-up revealed gradual recovery of Hb levels, reaching 11.9 g/dL after approximately 4 months, along with stable platelet counts and reticulocyte levels. Additionally, echocardiographic evaluations showed no signs of recurrent PVL (Figures 2A to 2F, Video 3).

## Discussion

We present the cases of 3 patients with PVL after TAVR that induced severe hemolytic anemia, which was successfully managed with percutaneous PVL plugging. Table 1 compares the baseline characteristics, laboratory findings, and procedural aspects of the 3 patients. These cases highlight the variable clinical presentations and diagnostic challenges associated with PVL-induced hemolytic anemia. Compared with mitral valve prosthesis–associated PVL, PVL in the aortic position atypically manifests with hemolytic anemia. The diastolic transvalvular pressure gradient across the aortic valve is significantly lower than the corresponding gradient between the left atrium and the left ventricle at the mitral valve position. This lower gradient is typically insufficient to generate the shear stress required to cause significant hemolysis through the PVL, which is the primary etiologic factor for hemolytic anemia. The occurrences in these cases demonstrate that there are additional fluid dynamic factors to consider beyond the driving pressure gradient.

**Table 1:** Demographic Characteristic, Laboratory Findings, Procedures, and Follow-Up

	Case 1	Case 2	Case 3
Demographic characteristics			
Age, y	77	69	74
Sex	Male	Male	Male
Laboratory findings at the time of admission			
Hemoglobin, g/dL	8.2	7.9	6.9
Platelets, × 10^3^/μL	343	163	142
Lactate dehydrogenase, U/L	1,395	1,275	2,616
Haptoglobin, mg/dL	<10	<10	<10
Schistocytes	2+	2+	1+
Reticulocytes, %	4.2	5.8	3.1
Pre-TAVR CT scan			
Aortic valve morphology	Tricuspid	Bicuspid type 0	Bicuspid type 1
Annulus area, mm^2^	471	549	440
Grade of calcification	Severe	Moderate	Severe
TAVR procedure			
TAVR valve type	Edwards Sapien 3 Ultra (Edwards Lifesciences)	Edwards Sapien 3 Ultra	TAV in TAV Edwards Sapien 3 Ultra/ Edwards Sapien 3 Ultra
TAVR valve size, mm	26	26	23/23
PVL post-TAVR	Mild to moderate	Mild	Mild
PVL closure procedure			
PVL closure device	Amplatzer Vascular Plug II (Abbott Vascular)	Amplatzer Muscular VSD Occluder	Amplatzer Vascular Plug II
PVL device dimensions, mm	10	8	10
Balloon dilatation, mm	True Balloon (BD Life Sciences), 25	True Balloon, 25 and 26	Not performed
PVL after procedure	Trace	Trace	None
Follow-up at 1 month			
Hemoglobin, g/dL	13.1	11.6	11.9
PVL at TTE	Trace	Trace	None

CT = computed tomography; PVL = paravalvular leak; TAV = transcatheter aortic valve; TAVR = transcatheter aortic valve replacement; TTE = transthoracic echocardiogram.

Two of the 3 patients did not present with typical symptoms of anemia or LV volume overload (asthenia, angina, dyspnea, orthopnea, or paroxysmal nocturnal dyspnea), with significant anemia revealed only during routine laboratory examinations. Diagnosing PVL-induced hemolytic anemia can be particularly challenging in the presence of complex multisystemic comorbidities. In 2 patients, underlying hepatobiliary and hematologic comorbidities complicated the diagnosis, especially in case 3, where PVL severity was only mild on echocardiographic imaging. All 3 patients demonstrated resolution of hemolytic anemia after percutaneous plugging, thus confirming the cause of the hemolytic anemia and demonstrating the efficacy of the intervention.

PVL remains a frequent and formidable complication of TAVR, and it may lead to LV volume overload and heart failure sequelae. Despite a substantial body of evidence highlighting the adverse prognostic implications of significant PVL following TAVR,^1^, ^2^, ^3^ there remains no general consensus on the management of this complication. Notably, the most recent European and American guidelines both cautiously recommend transcatheter closure of prosthetic valve PVLs exhibiting concomitant severe hemolysis (Class 2a Recommendation, Level of Evidence: B).^4^^,^^5^ However, the evidence underpinning these recommendations largely emanates from experience with surgical prostheses in the mitral and aortic position.^6^^,^^7^ Furthermore, insights into post-TAVR PVL closure remain somewhat limited, primarily sourced from retrospective registries characterized by relatively modest sample sizes.

One study specifically focused on transcatheter plugging of TAVR-related PVL, the PLUGinTAVI registry, which included 45 patients from several European centers. Of these patients, only 1 presented with hemolysis, and the rest had heart failure symptoms.^8^ This finding supports the notion that severe hemolytic anemia from post-TAVR PVL is a scarcely described phenomenon and most PVL closure is largely driven by patients presenting with heart failure symptoms or LV dilation from volume overload.

Transcatheter PVL closure remains a technically challenging procedure, marked by variable success rates and clinical outcomes contingent on the experience of the performing center and the chosen closure modality (comprising balloon post-dilation, plugging, or valve-in-valve techniques).^8^, ^9^, ^10^ Perhaps this highlights the need for closer investigation to delineate the PVL characteristics (ie, PVL location, orientation, shape and size, presence of native valve calcifications, and transcatheter heart valve implanted) that render each closure modality most efficacious. Our cases demonstrated a stepwise percutaneous approach to PVL closure. All patients underwent balloon dilatation (performed before the second TAVR in case 3) before we attempted to plug the PVL with an occluder device. High-quality intraprocedural imaging to re-evaluate the PVL after balloon dilation and before occluder release is paramount to successful closure. In line with this, recent findings underscore the critical role of successful PVL reduction in patients undergoing percutaneous closure, with patients achieving a reduction to mild or less PVL demonstrating a pronounced mortality benefit at 1 year.^10^ In cases of hemolytic anemia, the importance of complete resolution of PVL is likely more pronounced because hemolysis is possible even with mild PVL.

Two of the 3 patients in this case series had bicuspid anatomy and high surgical risk, the decisive factors in the decision to proceed with TAVR. Although bicuspid anatomy is associated with higher rates of post-TAVR PVL, the use of intraprocedural TEE in patients with bicuspid valves, as now routinely implemented at our center, may further optimize outcomes by allowing for the precise assessment and management of PVL during the index procedure.

PVL is a well-documented complication of TAVR, although here we present insidious clinical presentations of severe hemolytic anemia from TAVR-related PVL. Although the procedure remains technically challenging, requiring high-quality intraprocedural imaging and operator expertise, adequate reduction of the PVL led to the resolution of hemolytic anemia in these patients. Further research is necessary to substantiate these procedural benefits and to refine the guidelines on the basis of on larger and more definitive studies.

**Visual Summary:**
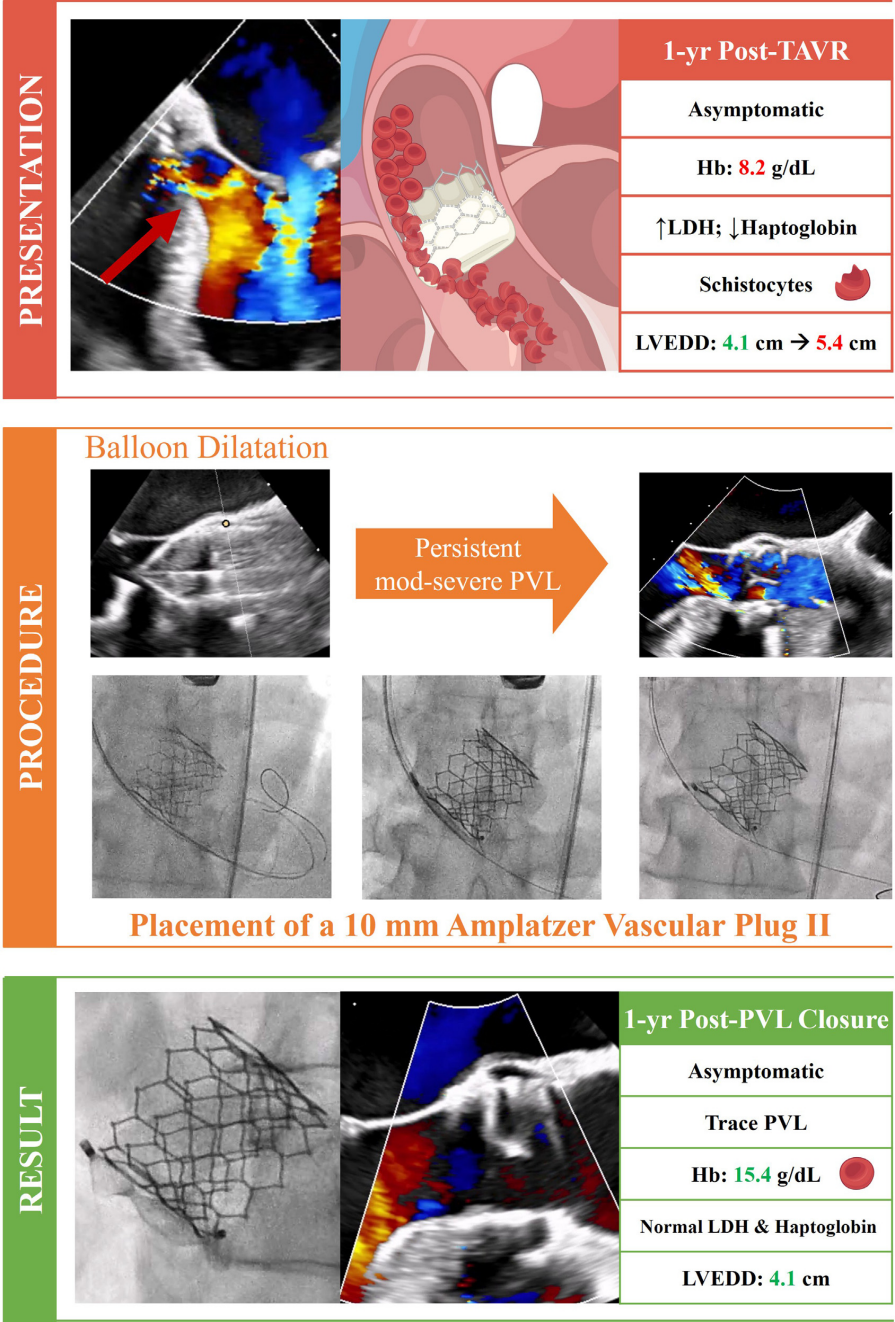
Graphic Representation of Case 1 Presentation, Transcatheter Intervention, and Final Result, Including 1-Year Follow-Up Hb = hemoglobin; LDH = lactate dehydrogenase; LVEDD = left ventricular end-diastolic diameter; mod = moderate; PVL = paravalvular leak; TAVR = transcatheter aortic valve replacement.

## Funding Support and Author Disclosures

Dr Scotti has served as a consultant for NeoChord and Edwards Lifesciences. Dr Echarte Morales has received support from Spanish Society of Cardiology through a mobility grant (SEC/PRS-MOV-INT 22/001). Dr Latib has served on advisory boards or as a consultant for Medtronic, Boston Scientific, Philips, Edwards Lifesciences, Abbott, and Ancora Heart. All other authors have reported that they have no relationships relevant to the contents of this paper to disclose.

## Appendix

Video 1 Case 1 Video Summary The paravalvular leak was worked up with transesophageal echocardiography, which showed moderate to severe paravalvular leak in the area of the noncoronary cusp. Paravalvular leak closure was first attempted with balloon post-dilation. This was unsuccessful because residual moderate to severe paravalvular leak was observed. Paravalvular leak closure was attempted with an Amplatzer Vascular Plug II (Abbott Vascular). Crossing the lesion was achieved with a hydrophilic wire. The distal disk of the Amplatzer Vascular Plug II was exposed from the 6-F Destination sheath (Terumo) and pulled back to the ventricular inlet of the paravalvular leak. The sheath was pulled back to allow the Amplatzer Vascular Plug II to expand within the paravalvular leak. The Amplatzer Vascular Plug II was then released. The procedure was performed under transesophageal echocardiography guidance. Before final plug release, transesophageal echocardiography was used to confirm adequate reduction of the perivalvular leak. The final result showed only a trace residual paravalvular leak. At 1-year follow-up the procedural result was maintained, showing a trace paravalvular leak.

Video 2 Case 2 Video Summary Video summary of case 2 showing the step-by-step echocardiographic evaluation and percutaneous management of this patient with significant hemolytic anemia from paravalvular leak. Follow-up imaging at 6 months showed only a trace paravalvular leak.

Video 3 Case 3 Video Summary Case 3 highlights an escalated interventional approach to paravalvular leak management. Redo transcatheter aortic valve replacement was first attempted, but it was unsuccessful in resolving the paravalvular leak and severe hemolytic anemia. Percutaneous plugging with an Amplatzer Vascular Plug II (Abbott Vascular) successfully reduced the paravalvular leak, and at 1-year follow-up follow no paravalvular leak was evident.
